# “How can a woman live without having a breast?”: challenges related to mastectomy in Ethiopia

**DOI:** 10.1186/s12885-023-11801-6

**Published:** 2024-01-11

**Authors:** Abigiya Wondimagegnehu, Solomon Teferra, Mathewos Assefa, Bradley Zebrack, Adamu Addissie, Eva J. Kantelhardt

**Affiliations:** 1 Department of Preventive Medicine, School of Public Health, College of Health Sciences, Addis Ababa University, Addis Ababa, Ethiopia; 2 Global Health Working Group, Institute of Medical Epidemiology, Biostatistics and Informatics, Martin-Luther-University, Halle, Germany; 3 Department of Psychiatry, School of Medicine, College of Health Sciences, Addis Ababa University, Addis Ababa, Ethiopia; 4 Department of Oncology, School of Medicine, College of Health Sciences, Addis Ababa University, Addis Ababa, Ethiopia; 5 School of Social Work, University of Michigan, Ann Arbor, USA; 6 Department of Gynecology, Martin-Luther-University, Halle (Saale), Germany

**Keywords:** Breast cancer, Breast surgery, Mastectomy, Refusal, Reasons, Ethiopia

## Abstract

**Background:**

Although mastectomy is the standard treatment modality for breast cancer patients in Ethiopia, our previous study revealed that one in five patients do not receive the recommended procedure, half due to patient refusal or lack of returning to the hospital. Therefore, this study aimed to explore reasons for refusing mastectomy and identify challenges among breast cancer patients in Ethiopia.

**Methods:**

An explorative qualitative study was conducted in four hospitals located in the towns of Woliso, Butajira, Hossana, and Assela. A total of 14 in-depth interviews (IDIs) and eight focus group discussions (FGDs) were held with breast cancer patients, patient relatives, and health professionals. Four semi-structured interview guides were used to facilitate the IDIs and FDGs. All recorded IDIs and FGDs were transcribed and translated verbatim and entered in NVivo 12 software. Emerging ideas were categorised and explained using an inductive content analysis approach.

**Results:**

Our participants reported that particularly elderly and very young women refuse to have mastectomy. The main reasons identified in this study were summarised into six themes: (i) fear of the surgical procedure, (ii) religious beliefs and practice, (iii) utilisation of traditional treatments, (iv) in relation to having a baby and breastfeeding their children (young patients often request to remove only the lump, leaving their breast tissue intact), (v) lack of awareness about the disease, and (vi) sociocultural factors and advice from the community that influence women, since breasts are considered an attribute of femininity, beauty, and motherhood. In addition, knowing someone who died after mastectomy emerged as a main reason for not having breast cancer surgery.

**Conclusions:**

High refusal rate for mastectomy has direct implication on increased breast cancer mortality. Hence, expansion of radiotherapy service is instrumental to initiate breast-conserving surgery as an alternative surgical procedure, especially for young women with early-stage breast cancer. Involving religious leaders, traditional healers, and breast cancer survivors could be an effective strategy to persuade newly diagnosed breast cancer patients. Addressing individual patient psychosocial needs and preferences may substantially improve retention of breast cancer patients in the health system.

## Background

Surgery is an important part of breast cancer treatment along with radiation and systemic treatments such as endocrine therapy, cytotoxic chemotherapy, targeted therapy, and immunotherapy [1]. Mastectomy and breast-conserving surgery (BCS) represent the two primary surgical options used to treat invasive breast cancer [2]. The survival benefit for undergoing surgery remains a well-known fact, as several studies found breast cancer patients who refused surgery had higher risk of mortality compared with those who had surgical treatment [3, 4].

In developed countries, having breast surgery is standard and according to a study done in the United States of America (USA), 95% of breast cancer patients underwent surgical treatment [5]. Not only therapeutic breast surgery, but contralateral prophylactic mastectomy (CPM) has also increased steadily over the last twenty years in women of all age groups and breast cancer stages [6, 7]. Genetic testing, pathogenic mutation of BRCA, strong family history, and suspicious findings on breast MRI were identified as the main reasons for its increment. As a result, most patients with unilateral breast cancer or even healthy women with high risk of breast cancer underwent CPM followed by immediate breast reconstruction [6, 7].

In contrast, frequency of breast cancer surgery in low- and middle-income countries (LMICs) remained very low even though it is the only treatment option available in most countries [8]. In Africa, limited resources for systemic therapy have contributed to the application of surgery as the primary modality for the management of resectable breast cancer [9, 10]. Even in some countries like Uganda, large areas of the country have no breast surgery at all and most of the surgical procedures are still being performed at the national referral hospital [11].

Among the various types of breast cancer surgeries, modified radical mastectomy (MRM) is the most common procedure in many African countries [8, 9]. According to a continent-wide review, about 85% of breast cancer patients undergo mastectomy because of the advanced stage of the disease [10] and reports from east Africa indicate that up to 99% of patients undergo mastectomy due to unavailability of other treatment modalities [9, 10]. Lack of or poor access to radiation facilities is the rate-limiting step for breast conservation in many African countries [8–10]. In sharp contrast, BCS is the preferred surgical procedure in developed countries and the percentage of breast cancer patients who had a mastectomy is often below 30% in most European countries [12] and 22.4% in the USA [13].

Nevertheless, mastectomy is considered culturally objectionable in sub-Saharan Africa (SSA), and let alone to choose CPM, the decision to have therapeutic unilateral mastectomy remains a big challenge for many breast cancer patients in the region. Therefore, compliance by patients vary from country to country and the refusal rates in countries like Eritrea and Cameroon are lower compared to Nigeria [10]. Inversely in western countries, refusal for further surgery such as breast reconstruction and oncoplastic surgery might be more common than refusing therapeutic mastectomies [12].

In Ethiopia, breast cancer is the leading cancer with an estimated 16,133 (20.9%) new cases and 9, 061 (17.5%) new deaths in 2020 [14]. Like other LMICs, there are limited diagnostic and treatment services in the country and the only available service for most breast cancer patients at lower-level hospitals is surgery [15]. Except endocrine treatment, systemic treatments are limited only in tertiary-level hospitals, which are mainly located in the capital or a few big cities [16, 17]. For instance, Tikur Anbessa Specialized Hospital was the only cancer centre that provided comprehensive cancer care including radiation treatment for the last couple of decades [17]. Recently, Jimma university hospital initiated radiotherapy services but still fell short of the international atomic agency recommendations of four machines per 1 million population [18]. There are only two radiotherapy machines for a population of 125 million, which makes breast-conserving surgery inaccessible and the standard for breast cancer in Ethiopia is MRM [16, 19].

Even though surgery is the only accessible treatment option in most peripheral hospitals of Ethiopia, many breast cancer patients do not even receive this service for several reasons [20, 21]. According to a study conducted in North Ethiopia, surgery was omitted in 32% of breast cancer patients because of an advanced stage of the disease with initial metastasis. MRM was the most common surgical procedure performed for 91% of breast surgeries done in Gondor Hospital [22]. Recently, a multicentre study in Ethiopia revealed that one in five patients did not receive the recommended surgical procedure, half due to patient refusal or lack of the patient returning to the hospital [16]. This is one of the missed opportunities leading to poor treatment outcomes and high mortality from breast cancer in the country.

Therefore, this study aimed to explore main reasons for refusing mastectomy and identify challenges in relation to having surgery among breast cancer patients in Ethiopia. Identifying those factors may enable the design of effective strategies to retain breast cancer patients in the health care system, which potentially improves adherence to subsequent treatments, enhance quality of life, reduce mortality, and improve overall survival. In addition, the economic benefit is far from reach compared to costs incurred to treat advanced cancer cases and through improving survivorship and productivity of breast cancer patients.

## Methods

### Study design and place

Exploratory qualitative study was employed to identify reasons for refusing mastectomy among breast cancer patients in Ethiopia. The study was conducted in four hospitals: St. Lukas Catholic Hospital Woliso, Negist Elleni Mohammed Referral Hospital Hossana, Butajira General Hospital, and Assela University Teaching and Referral Hospital. The hospitals are situated within a 130–250 km radius from the capital city, Addis Ababa. These hospitals and towns were purposively selected based on the different geographic directions from Addis; southwest, south, southeast, and east, and due to their potential for representing several Ethiopian populations located in two large regions, namely Oromia and Southern Nations, Nationalities, and Peoples’ Region (SNNPR).

### Study participants and sample size

In this study, fourteen in-depth interviews (IDI) and eight focus group discussions (FGDs) were conducted with a total of 70 participants (18 males and 52 females). We used a purposive sampling strategy to assure a broad variety of information. With regard to the actual participants recruited in our study, we intentionally selected both health professionals and cancer patients in order to gain the two different perspectives and also triangulate our findings. Even among the health professionals, we included clinical staffs working at the different levels of the health care system. Three IDIs with patient relatives and three IDIs with breast cancer patients who refused to have a mastectomy were held. Out of these three women whom we interviewed, one of them was 90 years old while the other two were 37 and 42 years of age. Of this, two of the women were married and had children but the other woman who was 37 years of age was not married and did not have children at the time of interview. In each town, two IDIs with health extension workers (HEWs) (community workers providing primary health care service at health post level which is the lowest health facility in the three health care tier system of Ethiopia) and two FGDs with breast cancer patients who had undergone surgery and health professionals (surgeons, oncology residents, head nurse, oncology nurse, midwife, maternal child health (MCH), gynaecologist, pathologist, general practitioner, and health officer) were conducted separately (Table 1). The participants were purposely selected, and an attempt was made to achieve the maximum variation of participants by including different age groups, rural and urban residency, gender, educational attainment, and interaction and provision of care for breast cancer patients.

### Data collection tools and procedures

Based on the objective of the study, four semi-structured interview guides were prepared to facilitate the IDIs and FGDs. Initially, all the interview guides were prepared in English and translated to Amharic (local language). The data was collected by two well-trained data collectors who had ample experience in facilitating qualitative data. On average, each IDI took around 21 to 49 min, while the FGDs stayed for a minimum of 58 min to a maximum of 103 min. Detailed field notes were taken during IDIs and one of the data collectors was taking notes while the other is moderating the group discussions. In addition, data collectors wrote reflexive memos immediately after completing each IDI and FGD. Daily debriefings on emerging thematic areas were done with the entire research team and the data was collected until we reached the theoretical saturation level. All FGDs and IDIs were audio-recorded, and notes were taken during the interviews and discussions. The data were organised and appropriately labelled immediately after each session. Subsequently, all recorded IDIs and FGDs were transcribed and translated verbatim after repeatedly listening to the recordings.

### Data analysis procedures

The data analysis was initiated simultaneously with the data collection process and a qualitative content analysis technique was employed. Daily debriefings on the newly emerging ideas were held with the entire research team, along with insiders who lived in those selected towns for a long period of time and who knows the community very well including the culture, language and social structure. All recorded observations and events during data collection were discussed and involvement of insiders in our debriefing sessions enabled us to verify the emerging ideas and properly interpreted the findings. It also supported us to understand the unique wordings, expressions and idioms revealed in our study. Once all transcripts were prepared by the data collectors, the principal investigator checked whether all IDIs and FGDs were properly transcribed and translated through listening to the audio recordings. All the recorded audios and transcripts were repeatedly reviewed to obtain an overall sense for and get familiarised with the data. Then, all transcribed documents, field notes, and reflexive memos were entered into NVivo 12 plus software. The analysis began by highlighting sentences of importance and dividing them into meaning units. Each document was coded line by line and the meaning units were condensed and labelled with short codes. Subsequently, similarities and differences between the codes were identified and categories were developed. Data triangulation was used to identify convergence of data obtained through IDIs and FGDs. Also through comparing and contrasting the reasons given by the patients themselves and their relatives and health professionals. Finally, emerging themes and subthemes were tested and revised through discussion with the team members and appropriate interpretations were given.

### Ethical clearance

Ethical clearance was obtained from the Research Ethics Committee of the School of Public Health and Institutional Review Board of College of Health Sciences, Addis Ababa University. Informed consent was obtained from all participants. Confidentiality and anonymity of the data was kept during data analysis and presentation of the findings. All methods were carried out in accordance with relevant guidelines and regulations in the Declaration of Helsinki.

## Results

The main findings of this study are organised into two themes. The first theme discusses refusal of mastectomy through given actual experiences of health professionals and patients. In the second theme, we identified several reasons why breast cancer patients refuse mastectomy, and these factors are further categorised in to six sub themes: (a) in relation to the surgical procedure, (b) due to religious beliefs and practices, (c) traditional medicine, (d) in relation to having a baby, (e) lack of awareness, and (f) sociocultural factors. The details of each theme and subthemes are described below.

**Table 1:** Characteristics of study participants, Ethiopia

Name of towns	No of IDI^*^	No of FGD^a^	Age range of participants	Male	Female	Role
Assela	2 IDI_with HEW^b^	2 (1 with HP^c^ & 1 with BCa^d^ patients	30–50	5	8	Surgeon, GP^e^, Oncology resident, oncology and psychiatry nurse, nurse head
Woliso	2 IDI_with HEW 1 IDI_with patient relative	2 (1 with HP & 1 with BCa patients	27–60	4	8	Surgeon, clinical nurse, surgical ward head nurse, psychiatric nurse
Hosanna	2 IDI_with HEW 1 IDI_ refused BCa pt	2 (1 with HP & 1 with BCa patients	35–55	4	12	Surgeon, Pathologist, Surgical ward nurse, Psychiatric nurse MCH^f^ focal, Midwife
Butajira town	2 IDI_with HEW 2 IDI_Refused BCa pt 1 IDI_Patient relative 1 IDI_Patient relative_refused	2 (1 with HP & 1 with BCa patients	27–90	5	14	Surgeon, pathologist, gynaecologist, surgical ward nurse, midwife, health officer
**Total**	**14**	**8**	**27–90**	**18**	**52**	

^*^ IDI: in-depth interview, ^a^ FGD: focus group discussion, ^b^ HEW: health extension workers, ^c^ HP: health professionals, ^d^ BCa: breast cancer patients, ^e^ GP: general practitioner, ^f^ MCH: maternal and child health

### Women refusing mastectomy

Our results found that breast cancer patients in Ethiopia refuse to have a mastectomy for several reasons. This idea was reflected in all FGDs and IDIs conducted with both cancer patients and health professionals. Most of the health professionals involved in the FGDs reported that many breast cancer patients refuse to have a mastectomy and they usually struggle to convince them. For example, a surgeon at Hosanna hospital mentioned that *“…there is resistance to have the surgery. Most of the time, they [breast cancer patients] accept the surgery after having two or more visits. For that matter, there are also others who refuse at all and return back to their home.* (FGD_ HP_Surgeon_Hossana)

*“The challenge starts during admission. Since it is removing a body organ, most women do not accept this. They are afraid of hearing about mastectomy, they do not accept it easily. They often agree after taking much time and through several attempts to convince them. Most of the time, they went back to their home and spent several months until they return back to us.”* (FGD_HP_Nurse_Woliso).

Some of the patients confessed that they refused initially and later decided to have the surgery. We also encountered breast cancer patients who still continue to resist surgery.

*“No way, I am not going to be operated. Oooo, ere ete I do not want to be operated. I have St. Michael. SIBHAT LAB! In the name of the father, the son and the holy spirit. May you forgive me St. Gebriel. I will never be operated!”* (IDI_Refused BCa_Butajira).

Even though the challenge was expressed in all study sites, the extent of refusal varies from place to place. For instance, in Woliso, the surgeon reported that most of the patients resist or totally refuse to have the surgery and return back to the health facility at an advanced stage where only supportive care is given.

*“A lot of patients refuse mastectomy and usually get lost and will never come back. For example, last week, I sent one woman to Addis Ababa for diagnosis and came with her result. I was waiting for her and preparing a place to do the surgery, but she disappeared. So, we do not know where she is now. She is lost! Umm… from all diagnosed cases only less than 50% had mastectomy. But most discharged refusing it.”* (FGD_HP_Surgeon_Woliso).

*“Most of the women who refused the surgery returned back to us after the disease advanced with a fungated breast and those women have died by now and only few are surviving.”* (FGD_HP_Surgeon_Woliso).

On the other hand, the surgeon from Butajira reported that patients often do not refuse to have surgery, rather they request to get priority. *“This was actually the only woman who refused surgery, but most patients want to have the surgery immediately after diagnosis. They prefer to get priority for surgery and do not refuse so far.”* (FGD_HP_Surgeon_Butajira) However, two of the three patients interviewed in this study for refusing mastectomy were from Butajira town.

### Who most refuses

Concerning specific subgroups who most refuse, the health professionals reported that young women (mostly referring to those who are below 40 years of age) who are not married and do not have children or with small babies often refuse mastectomy. Again, very old people also refuse to have the surgery due to hopelessness and fear of the procedure.

*“…the most resistance is coming from either very old or very young women. Especially those who are below 30 refuse to have mastectomy. There is such a kind of resistance from them. It is psychologically difficult to accept mastectomy for the younger ones. It would have been good if we have breast conserving surgery for them.”* (FGD_HP_Surgeon_Hossana).

### Reasons for refusal—afraid of the surgical procedure

In this study, several factors were identified for not having a mastectomy and a major one was fear of the surgical procedure. Many patients mentioned that they are afraid of the surgery, assuming that the anaesthesia or the surgery itself may kill them. This reason was mainly reflected from elderly people, and health professionals also reported trying to convince such patients through counselling.

*“I was simply afraid of it and never thought that I will be healthy after having a surgery.”* (FGD_Refused BCa_Butajira).

*“But for few of the patients the reason is due to fear of the surgery. They think they may die from the surgery. For those who are afraid of surgery, it is possible to advise them for a short period of time and convince them.”* (FGD_HP_Nurse_Woliso).

### Knowing someone who died after mastectomy

In addition to fear of the procedure, some participants also mentioned that they do not trust in the effectiveness of the surgery and have encountered some patients who died even after breast removal.

*“I am afraid because I know a woman whose breast was removed and died after one year with a lot of pain. It has been so long since this happened, but I always remember her. She always comes into my mind whenever they advise me to be operated. I am not afraid of surgery since I was operated before for another problem. But since I saw that woman who died after her breast was removed, I consider that my fate will also be like her. I will die if I am operated. That’s why I am afraid of the surgery.”* (IDI_Refused BCa_Hossana).

Some of the participants mentioned that they prefer to take oral medications and only treat the cancer with medications rather than being operated. Surgery is considered the last option after exhausting other treatments. They have also reflected that they are willing to take any form of medication (tablets or injections) in lieu of the operation.

*“Whatever it takes, I can take that kind of drug. I do not care whether it will be taken for a short or long time. But if I get it, I will use it, but I do not want to be operated.”* (IDI_Refused BCa_Butajira).

### Why the whole breast?

The surgeons in our FGDs reported that patients usually resist having a mastectomy because they request to remove only the mass and it is difficult to convince them why the whole breast should be removed. Especially younger women and those with a painless small mass do not agree to remove the whole breast.

*“The challenge is not with totally refusing the surgery. They choose among the types of surgery to be done. They want you to remove only the diseased part or the mass/lump which means breast conservative therapy. So, after agreeing to have the surgery, they consult you “Why don’t you only remove the cancer/lump rather than removing the whole breast?"”* (FGD_HP_Suregon_Butajira).

### Due to religious beliefs and practices

This study revealed that there is a belief in the community that cancer cannot be cured by medical treatment. Due to this and hopelessness, cancer patients prefer to visit religious places. Then, after spending several months in those places, they will return to the health facilities at such an advanced stage that they can not receive curative service, only supportive care.

*“For example, there was a woman here who refused to have a mastectomy and she went to a religious place. She thought that she would be cured by religious things/faith. Therefore, there is such a belief in this area.”* (FGD_HP_Nurse_Butajira).

Almost all the cancer patients involved in this study mentioned that they have visited religious places and tried different religious rituals to get cured. Some of the patients mentioned that they are simultaneously using both the medical treatment and religious practices, while others totally disagree with the medical plan and only took religious treatments such as prayers, holy water, emnet (some powder to be applied on their body), and duwa (prayer in Islam religion). Especially among those breast cancer patients who refused to have a mastectomy, agreeing to remove their breast demonstrates a lack of faith in God or the angels. Going to the health facility and removing their breast means not trusting God and going above His supernatural power. For instance, one of the women who refused to have a mastectomy said:

*“I prefer to die now rather than losing my breast. I have the word of God and I promised to God that " You are the one who created all my body parts, and you will give me back my body as it used to be. I will not be operated unless it is difficult for you [referring God]” I have a strong faith in God. So, I believe that there is nothing above the power of Him. I have told them [referring to the health professionals] this from the beginning and they were telling me to think about it. But I told them as I have already decided a long time ago and there is nothing that I am going to think about anymore.”* (IDI_Refused BCa_Butajira).

A very old breast cancer patient who refused to have a mastectomy even described it as a sin to agree to have the surgery at this age.

*“For what reason? No way, I do not want to have surgery on any part of my body. hoooo, I will never be operated as long as St George is alive, St, Mary and St. Michael are alive. I already decided on this. I swear to myself. My sisters also advised me to be operated but I gave my witness to God. I am going to be a Monk so I can not be operated. In the name of the father, the son and holy spirit (saying it with sign). Oooo, ere ete I do not want to be operated.…. It is a sin. I do not know whether I will die now or tomorrow. May you forgive me St. Gabriel. I will never be operated!”* (IDI_Refused BCa_Butajira).

Our results also identified pressure from religious leaders and community members to stick to religious practices rather than medical care. Moreover, it is highlighted that while there are many religious places nearby, cancer patients tend to travel very far to get healed, and spend a lot of money and time visiting different monasteries.

*“One of the major challenges these days is, there are several religious practices, especially prayer is very common around here. There are a lot of prayer houses everywhere. They [religious leaders] even order them [the patients] to stop their medication and follow only the religious activity. Umm… the other woman also said, " I am not going to have the surgery. The servant of God told me that God will remove this disease from me, and I should not allow a knife to be put on my body” Therefore, she refused to have the surgery.”* (FGD_HP_Nurse_Hossana).

### Due to traditional medicine

One of the identified reasons women refuse to have surgery was a strong belief in the community; that traditional medicine is more effective than medical care or surgery. Most people in the community believe that cancer can be cured by different herbals and the patient can survive without losing their breast or other body parts.

*“The community perceives that cancer cannot be cured by medical treatment. Yaw… they say, it can only be cured by traditional medicine.”* (FGD_BCa_Assela).

With few exceptions, most patients we interviewed tried different traditional medicines suggested by community members. Even those who already started medical care mentioned that many people were advising them to take traditional medicine rather than strictly following medical care.

*“Traditional medicine is available anywhere. At the beginning, people were advising me to use traditional medicine and not to go to the health facility. A lot of people were advising me this in my village, but I came to the hospital without accepting their idea.”* (FGD_BCa_Hossana).

### Because they want to have a baby

The other interesting finding of this study is that women will refuse to have a mastectomy if they want to have a baby and breastfeed their child. This problem is more prominently reflected among young women who are not married and want to have a baby. Health professionals reported that they face challenges in convincing this particular age group to have surgery, and the greatest challenge is the financial constraint of formula milk for their baby.

*“About the mastectomy, especially for the young women, it is very difficult to accept the surgery since it has its own effect on having a baby. It is totally challenging and has a lot of problems. That could also be one of their reasons for refusal.”* (FGD_HP_Nurse_Hossana).

*“Actually, those women who are in the childbearing age are afraid of having the surgery since they want to breastfeed their kids. But still, we advise them to have the surgery because they can use one breast for breastfeeding. Once, I was in a dilemma since she cannot afford to buy formula for her baby. So, it is difficult to advise her not to breastfeed and only give formula.”* (FGD_HP_Nurse_Woliso).

Additionally, a few participants mentioned that breasts have a strong meaning for women and is a representation of their femininity. One of the breast cancer patients said about losing her breast, *“So I always say to God, ‘You created me with full body parts and please do not make my breast diseased before I give birth and before I breast feed my children. You are the one who gave me my breast, it is my feminine symbol’.”* (IDI_Refused BCa_Butajira).

Similarly, another woman who refused to have a mastectomy described her feelings on the relationship between her breasts and the bond with her children. *“Wuyyyyyyyyyyy (shouted) Gebriel help me please!! How can I remove my breast which nine of my children fed? Never! It is better to remain sick and die with it rather than losing my breast”.* (IDI_Refused BCa_Butajira)

### Due to lack of awareness

In general, lack of awareness about breast cancer, particularly in relation to its clinical manifestation, treatment modalities, and prognosis was one of the main reasons why many women refuse to have a mastectomy. Especially at the early stage, where it mainly presents with a small painless mass, many women consider it a simple illness that will subside by itself. Therefore, many women prefer to keep the mass, as long as it is not painful and does not hinder their routine life. For instance, a surgeon from Hosanna hospital said:

*“Its presentation is usually small breast mass/ lump. The scenario for prostate, gastric and colonic cancer is totally different in which most patients present with obstruction. So, they are not in a position to refuse the surgery since they need relief. But this does not apply for breast cancer. A small painless mass is a concern for us, but most patients do not realize this at the beginning. To be honest, most of these patients return back and beg us to do the surgery after the swelling is burst and fungated.”* (FGD_HP_Surgeon_Hossana).

### Sociocultural factors and misconceptions

Many respondents mentioned that they were advised by community members not to have surgery. Even those who already had a mastectomy stated that they were told by their family members and neighbours not to have surgery, and even went so far as to advise them not to visit hospitals anymore but rather take traditional medicine or go to religious places.

*“As for me, I went back to my home fully convinced to be operated on Monday. But all of my family members and neighbours were not happy about it. None of them were willing to have the surgery, none of them. People around me were telling me not to have the surgery. They said, “How can you live without having a breast? How can a woman live without having a breast? She can not live at all, she will die” So, it’s better not to have the surgery and stay alive. A looottt… of people were warning me as I will die if my breast is removed.* (FGD_BCa_Woliso)

The caregivers we interviewed also stated that they faced such challenges from community members while they were struggling to convince their relatives. For instance, a son of a breast cancer patient who refused to have a mastectomy said:

*“She is really afraid of the operation. Because the community advised her not to be operated and as she may die if the surgery is done at this age. Since she is very old, she accepts what people said.”* (IDI_Son of Refused BCa pt_Butajira).

The other interesting findings of this study were community misperceptions about having a mastectomy. For instance, some people assume that cutting their breast might aggravate the illness and disseminate the cancer to other parts of the body and cause death. Others consider this surgical procedure as disability and prefer to die having all their body parts.

*“So, God created me with full body parts, so I do not want to miss any of my body parts while observing with my naked eyes.”* (IDI_Refused BCa_Butajira).

*“They think that the disease may reoccur if the surgery is done. People assume that, if this organ is removed, there is a probability of refilling it. Because of this, they say, “It is better if I die as I am complete rather than having the surgery.””* (IDI_HEW_Woliso).

## Discussion

This study revealed that many breast cancer patients in Ethiopia refuse to have a mastectomy, especially young and very old women, and often return to the health facility much later, when surgery is impossible. This finding is consistent with a study conducted in Rwanda that reported that 15.7% of breast cancer patients either refused the surgery or disappeared without receiving the recommended operation [23]. Similarly, a prospective multi-country study revealed that treatment refusal contributed to not initiating the recommended treatment. A high refusal rate (38%) was reported in two Nigerian hospitals, while almost all (98.7%) women in Namibia had initiated the treatment within one year of diagnosis [24]. Another study from Nigeria reported a high refusal rate, with only 32% of breast cancer patients having had the recommended operation [10]. Previous study conducted in eight hospitals located in southwestern Ethiopia also revealed that one in five cancer patients did not receive the reccomended surgical treatment; half due to refusal while the remaining did not return back to the health facility on their appointments [16].

In contrast, removing the breast is not considered as impactful in developed countries and the refusal rate is minimal. A study from the USA reported that out of 531,700 breast cancer patients identified in the Surveillance, Epidemiology, and End Results (SEER) database, only 0.64% refused surgery [25]. Another study using the same database from 2010 to 2015 found that 3.56% of a total 13, 618 patients with stage IV breast cancer refused the recommended primary tumour surgery [26], and of the 5,860 male breast cancer patients identified, only 0.9% refused surgery [3]. This huge difference in refusal rate between high- and low-income countries could be explained by lack of knowledge about the disease, low educational and economic status, and difficulty in accessing health services in LMICs [4]. On the other hand, availability of immediate breast reconstruction, improvement of postoperative aesthetic results, and reimbursement plays an important role in whether patients have therapeutic or prophylactic mastectomy in western countries [6, 7].

Our results identified that young women who are not married and do not have children often refuse mastectomy. Similarly, a qualitative study in Nigeria revealed that undergoing mastectomy at a young age may interfere with quality of life and overall accomplishment. These ideas may be responsible for fearing mastectomy, which makes young women delay or refuse treatment [27]. In previous studies, single or widowed marital status were found to be associated with refusal of surgery [4, 25]. The desire for future marriage, pregnancy, and breast feeding their children influence the treatment decision of many young women. In contrast, around one-third of patients in Uganda who received surgical treatment was below the age of 40 years and more than half of them were below 50 years [11]. This variation could be explained by the fact that the study did not report the refusal rate and only described the age of breast cancer patients who underwent mastectomy within one year, and it is known that most breast cancer patients in SSA are below the age of 40.

A high refusal rate for mastectomy among young women has a great implication since most breast cancer patients in Ethiopia and SSA are of childbearing age [8, 10, 16]. Basically, BCS is recommended as a local treatment for young women with early-stage breast cancer [28]. Given the absence of survival difference between MRM and BCS, the patients in developed countries are ultimately the ones who decide their preferred surgery, barring any contraindication [7]. For this reason, many patients who want to preserve their breast tissue decide to have BCS since radiation treatment is widely available in developed countries [29]. But the situation in LMICs does not allow young women to choose among the two surgical procedures and it is mainly the decision of the surgeon. In Ethiopia, most surgeons are forced to avoid BCS and instead perform mastectomy in most breast cancer patients, regardless of the stage at diagnosis [30]. Scarcely available radiotherapy services, with more than a year wait time, increases the risk of tumour recurrence and threatens the life of patients who have BCS. For this reason, MRM remains the standard surgery in Ethiopia today [17, 19]. This challenge was clearly reflected in our study, as many young breast cancer patients requested to have only the lump excised—not the entire breast—and this was one of their reasons for refusal and seeking alternatives. As evidence shows, the refusal group was associated with a poorer prognosis in overall survival compared to the surgery group [3]. This indicates that availing BCS should not be considered a luxury, but rather a mandatory alternative treatment option for young breast cancer patients in Ethiopia. Despite the administrative and technical challenges causing delay in the expansion of radiotherapy service in seven cancer centers in Ethiopia, Jimma university hospital recently started the service and became the second cancer center next to Tikur Anbessa Specialized Hospital [19]. However, having only two radiotherapy machines for a population of 125 million is still below the standard to consider BCS as an alternative surgical procedure for breast cancer patients in Ethiopia. Hence, efforts in expanding radiotherapy services all over the country requires due attention.

Our results identified that not only young women refused mastectomy, but older women also refused for various reasons. One of our participants who resisted having a mastectomy after repeated advice from health professionals and family members was over 90 years old. This finding is in line with a review of 22 studies done in USA, Asia, and Europe, which reported that breast cancer patients older than 70 years were more likely to refuse treatment, with the main factors including unmarried status, non-white race, female gender, and having government or no insurance [4]. Another quantitative study also identified that older age (≥ 65 years) was one of factors for refusal of mastectomy among male breast cancer patients [3]. In contrast, women undergoing mastectomy in China were older and more likely to be married and have at least one child [31].

Interestingly, all the refusal patients we interviewed were willing to take alternative treatments, either in the form of tablets or injections. Similarly, a case study of a 74-year-old woman in South Korea who refused mastectomy reported that a modified treatment process, including non-surgical primary therapies, minimised surgery, and close follow-up was successful, without signs of recurrence or metastasis after eight years from diagnosis [32]. In such conditions, endocrine and radiation therapy were chosen as treatment alternatives. A meta-analysis demonstrated that primary endocrine therapy (pET), even as monotherapy, has comparable response rates to neoadjuvant chemotherapy in patients with oestrogen receptor (ER) positive tumours, and with considerably lower toxicity [33]. Consistently, a retrospective study in South Africa revealed that pET can be a viable alternative for those patients over 70 years of age [34]. Not having an alternative modified treatment in LMICs is one of the missed opportunities to address those elderly patients who want to optimise all medical treatments except the surgery. Given considerably lower toxicity and relative ease of administering oral therapies in comparison with other systemic treatments, pET can be considered an effective approach in LMICs [1], particularly in a country like Ethiopia where radiation treatment is limited and more than 65% of breast cancer tumours are ER positive [35].

Knowing someone who died after having a mastectomy was one of the reasons identified in this study. This is probably because the actual intent of the performed surgical procedure was not properly communicated to patients and relatives, particularly for those procedures done with the intention of palliation. The other possible explanation could be, as with other surgical procedures, the community may expect total cure following surgery, even without receiving other systemic treatments. According to the Cochrane review, it is not possible to make definitive conclusions on the benefits and risks of breast surgery associated with systemic treatment for women diagnosed with metastatic breast cancer, and it was recommended that the discussion and decision to perform breast surgery should be individualised and shared between the physician and the patient [36]. Therefore, it is unknown whether surgeons were performing operations without ruling out distant metastasis since imaging and other diagnostic modalities are limited in peripheral sites of Ethiopia. The other reason for high mortality after mastectomy could be due to lack of systemic treatments; most likely these patients did not receive either chemotherapy or endocrine treatment after having the operation. Our study from rural Ethiopia showed that nearly all patients died within three years after surgery without systemic treatment [37]. Therefore, as is clearly shown in a Nigerian study [27] involving breast cancer survivors, this could play an important role in convincing newly diagnosed women to accept mastectomy in addition to professional counselling.

One of the important issues that emerged in refusal of mastectomy among breast cancer patients was seeking alternative treatments such as traditional medicine and religious practices. A previous study among breast cancer patients in Addis Ababa also identified that utilisation of traditional medicine was a significant factor in advanced stage presentation [38]. A similar finding was reported by a multicentre study conducted in three sub-Saharan countries wherein women who believed in traditional medicine and spiritual healings were eight times more likely not to have received conventional breast cancer treatment, including surgery [24]. Not only in African countries, but traditional medicine was also mentioned as one of the factors for delay in presentation in Singapore [39]. The easy accessibility of traditional and alternative medicine locally is a double-edged sword. Patients often choose to subscribe to alternative medicine, believing in its curative effect, thus delaying presentation and also initiation of treatment.

Patients perceive and experience illness, care, and death according to their culture, values, beliefs, life experiences, and meaning of life. Thus, it is argued that spirituality, culture, and the socioeconomic status may influence patients’ healthcare decision-making [4]. In this study, some breast cancer patients mentioned that people in the community were advising and frightening them against surgery, saying that they will not be considered a woman after losing their breasts. This is because breasts are presumed to be an attribute of femininity, maternity, and sexuality [30]. A qualitative study in Nigeria explored the perception that breast removal results in loss of femininity and womanhood, and that a woman without breasts is considered a man [27]. These community perceptions uniquely expressed in Africa can be partly explained by low disease awareness, women’s lack of economic dependence and decision making power, and strong attachments to motherhood and breastfeeding.

Our study reflected that breasts have special meaning, especially for elderly women in relation to motherhood and expression of attachment with their children. Therefore, they refused to have a mastectomy because of fear of losing this bond and considered it abandonment of their children. Similarly, a qualitative study in Sweden reported that breasts mean femininity, beauty, attraction, and motherhood [40]. After mastectomy, it was difficult for some women to accept the situation, as they felt they lost their femininity and considered themselves disabled or incomplete. Some studies showed that a mastectomy negatively affected a woman’s body image, as well as the relationship with their husband [27, 30, 41].

### Strengths and limitations

One of the strengths of this study is the inclusion of breast cancer patients who refused to have a mastectomy, which enabled us to understand their actual reasons for refusal. Use of both IDI and FGD data collection techniques enhanced the triangulation of our findings. Moreover, inclusion of health professionals ranging from community health workers to specialists supported us in capturing the whole situation and realising the different perspectives from both patients and health professionals. The findings of this study are limited to the geographic area covered, but we see that there are many themes also found in other literature. All interviews were conducted in the official local Amharic language, so possible nuances present in other local languages were not captured. Due to the rareness of the case, we could not include male breast cancer patients in our study and the health professionals reported as they didn not observe major challenges or resistent from male patients.

### Clinical significance

First, to meet more patients’ needs, especially young women with early-stage breast cancer, BCS should be made more available. Expansion of radiotherapy services is already ongoing, with a second site providing this service. Clear guidelines for BCS are needed to justify the additional radiation time to ensure BCS for those patients who have curative options and would otherwise abandon therapy altogether.

Second, in case of elderly patients who are not suitable for surgery, effective non-surgical primary therapies such as a combination of endocrine and radiation therapy should be considered.

Third, concepts and information must be tailored to the patient´s understanding. Fear about surgery, not understanding the reasons for mastectomy, and lack of awareness about disease severity are important points of agreement for undergoing such complex treatment modalities. Addressing individual patient psychosocial needs and preferences is also a key element for the retaining them in the health system.

Fourth, a high proportion of patients in LMICs are below the age of 40 and still of childbearing age. Conception following anticancer therapies is a key component to be considered. Therefore, health professionals should be conscious of this and try to build trust with these patients to facilitate open discussion about their personal life, future plans, and family size. Training on onco-fertility counselling might be useful.

Fifth, awareness creation programs are crucial in both religious places and through collaborative efforts with traditional healers, religious leaders, and influential people. Involving breast cancer survivors could also play an important role in persuading newly diagnosed breast cancer patients. Bringing these to the forefront and establishing patient support groups may have substantial impact. Guidelines on how to manage young breast cancer patients in Ethiopia may assist surgeons in decision-making, to include accessible adjuvant treatment. It is also recommended for future researchers to explore the lived experience of breast cancer patients after having mastectomy and involve male breast cancer patients in order to see the other dimension of the challenge from their own perspective.

## Conclusion

Many breast cancer patients in Ethiopia, especially young and elderly women, refuse to have a mastectomy. In this study, six main reasons for refusal of mastectomy were identified. These include fear of the surgical procedure itself, arguing for excision of just the mass and not the whole breast, and searching for alternative treatments such as religious rituals and traditional treatments. Particularly for young women, they often refuse in relation to having a baby and breastfeeding. Low awareness about the disease, especially its severity and prognosis, also emerged as a primary reason for mastectomy refusal. Moreover, sociocultural factors in the community, especially that of considering breasts a symbol of femininity and motherhood, influence women not to have the surgery. Poor survivorship stories after mastectomy also contributed to high refusal rate in the country.

## Data Availability

The datasets used and/or analysed during the current study are available from the corresponding author upon reasonable request.

## Abbreviations

BCS: Breast-conserving surgery
CPM: Contralateral prophylactic mastectomy
ER: Estrogen receptor
FGD: Focus group discussion
GP: General practitioners
HEW: Health extension workers
HP: Health professionals
IDI: In-depth interview
LMICs: Low- and middle-income countries
MRM: Modified radical mastectomy
PET: Primary endocrine treatment
QCA: Qualitative content analysis
SEER: Surveillance, Epidemiology, and End Results
USA: United States of America
